# Safety and Tolerability of Plasma Exchange and Immunoadsorption in Neuroinflammatory Diseases

**DOI:** 10.3390/jcm9092874

**Published:** 2020-09-05

**Authors:** Johannes Dorst, Frank Fillies, Jens Dreyhaupt, Makbule Senel, Hayrettin Tumani

**Affiliations:** 1 Department of Neurology, University of Ulm, 89081 Ulm, Germany; frank.fillies@uni-ulm.de (F.F.); makbule.senel@uni-ulm.de (M.S.); hayrettin.tumani@uni-ulm.de (H.T.); 2 Institute for Epidemiology and Medical Biometry, University of Ulm, 89081 Ulm, Germany; jens.dreyhaupt@uni-ulm.de

**Keywords:** therapeutic plasma exchange, immunoadsorption, neurological diseases, multiple sclerosis, chronic inflammatory demyelinating polyneuropathy

## Abstract

Plasma exchange (PE) and immunoadsorption (IA) are frequently used for treatment of various autoimmune-mediated neurological diseases, including multiple sclerosis (MS), chronic inflammatory demyelinating polyneuropathy (CIDP), and Guillain–Barré syndrome (GBS). Although both methods are generally regarded as well-tolerated treatment options, evidence for safety and tolerability is low for most indications and largely relies on small case series. In this study, we retrospectively analysed adverse events (AEs) and laboratory changes in 284 patients with various neurological indications who received either PE (*n* = 65, 113 cycles) or IA (*n* = 219, 435 cycles) between 2013 and 2020 in our Neurology department. One standard treatment cycle for PE as well as IA consisted of five treatments on five consecutive days. During every treatment, the 2.0–2.5-fold individual plasma volume (PV) was treated in IA, while in PE, the 0.7-fold individual PV was replaced by human albumin solution. Overall, both methods showed an excellent safety profile; no deaths of life-threatening adverse events were recorded. Severe AEs (corresponding to grade 3 on the Common Terminology Criteria for Adverse Events grading scale v5.0) including three patients with sepsis, one pneumonia, and one pneumothorax were present in 5/435 IA cycles (1.1%); in the PE group, no severe AEs were recorded. Furthermore, although advantageous tolerability is generally considered the main advantage of IA over PE, we found that overall frequency of AEs (including grades 1 and 2) was higher in IA (67.1% of all cycles) compared to PE (35.4%; *p* < 0.001). The low incidence of AEs in PE might be caused by the lower PV exchanged during each treatment (0.7-fold) compared to previous studies which predominantly exchanged the 1.0–1.5-fold PV. In order to verify this hypothesis as well as confirming the efficacy of this lower-dosed scheme, prospective studies comparing different treatment regimens are needed.

## 1. Introduction

Plasma exchange (PE) and immunoadsorption (IA) are used in various autoimmune-mediated neurological diseases in order to remove autoimmune antibodies and other pathological constituents from the patients’ blood. Currently, indications include multiple sclerosis (MS), myasthenia gravis, autoimmune encephalitis, chronic inflammatory demyelinating polyneuropathy (CIDP), Guillain–Barré syndrome (GBS), and many others. Although PE constitutes the standard technique for most diseases, IA is increasingly recognized as a more specific alternative and generally appreciated for its potentially advantageous safety profile. However, safety and tolerability of both methods have rarely been directly compared under standardized, monocentric conditions.

Originally, both treatment options primarily aimed at removing auto-antibodies from the blood, although various additional immune-modulating mechanisms like up- and downregulation of anti- and pro-inflammatory interleukins have been discussed [1]. Substantial methodological differences have to be considered which may affect efficacy as well as safety. Since in PE the plasma is removed and substituted by a volume replacement solution (human albumin or fresh frozen plasma (FFP)), all circulating proteins are removed, including coagulation factors. In contrast, IA relies on adsorbers which selectively bind human immunoglobulins (Ig) while largely sparing other plasma proteins; the processed plasma is led back to the patient, and no replacement solution is needed. Theoretically speaking, these factors should favor IA in terms of adverse events (AEs), while on the other hand the preservation of pro-inflammatory cytokines and other pathogenetically important proteins may weaken its efficacy dependent on the specific immunology of the respective disease, which is however, not fully understood in many cases. Furthermore, it has been shown that even in IA other plasma proteins are also affected which might explain its efficacy in diseases which are not regarded to be primarily antibody-mediated [2].

Apart from the method itself, specific techniques and treatment regimens have to be taken into account when assessing efficacy and safety of PE and IA. Various regenerable (protein A, recombinant proteins) and non-regenerable (tryptophan, phenylalanine) IA adsorbers are routinely used in clinical practice which feature different binding characteristics with regard to immunoglobulin classes, subclasses, and other plasma proteins [3,4]. For example, protein A adsorbers have a stronger binding affinity to IgG compared to IgA and IgM [3]. Furthermore, various treatment regimens can be applied for PE and IA with regard to number and frequency of treatments as well as the plasma volume (PV) treated during each session. Usually, 5–7 treatments are performed in both PE and IA, while treatment frequencies vary between daily and 2-day applications depending on fibrinogen levels. In IA, processing of the 2.0–2.5-fold individual PV constitutes the standard [5], which allows a daily treatment regimen for regenerable protein A and recombinant protein adsorbers, while a two-day treatment regimen with fixed PV (usually 2 or 2.5 L) is usually applied for non-regenerable tryptophan and phenylalanine adsorbers (due to loss of fibrinogen) [6,7,8]. In PE, various regimens with different PVs have been used. For example, the original randomized controlled trial (RCT) which built the foundation for the use of PE in MS applied 7 treatments within 14 days, exchanging the 1.1-fold PV during each session [9], while a more recent RCT showed that a daily treatment regime with 5 sessions and replacement of the 0.7-fold PV during each session was also effective [5]. Importantly, across all neurological indications there are no RCTs which directly compare different treatment regimens, and only few regimens have been tried; therefore, it seems very likely that the optimal regimen with regard to efficacy and safety has not yet been found.

Furthermore, specific peri- and intra-procedural measures vary between centers. In order to prevent blood in the extracorporeal circuit from clotting, heparin and/or citrate are most commonly used which carry various potential complications like heparin-induced thrombopenia and hypocalcemia. Some centers replace immunoglobulins after each treatment in order to account for the immunodeficiency induced by the therapy, while others rely on the periprocedural prophylactic administration of antibiotics. For all these measures, no reliable evidence exists. 

Previous studies comparing PE and IA with regard to safety and tolerability in neurological diseases predominantly reported either no differences [6,8,10], or advantages for IA [11,12]. Since PE is unspecific, various complications due to loss of coagulation factors and other plasma constituents have been reported such as thrombosis, bleeding, hypotension (due to volume-shift), and sepsis [13,14]. Furthermore, the need of a volume replacement solution carries the risk of severe allergic reactions [13]. Life-threatening complications have been reported in 0.12% of patients [14], and a higher risk of adverse events in patients with neurological diseases compared to non-neurological diseases has been described [13]. On the other hand, IA has repeatedly been described as a safe and well-tolerated procedure [3,4,15]. Two studies in myasthenia gravis found that side effects were reduced in IA compared to PE [11,12]. In MS, the majority of studies did however not report any differences between IA and PE with regard to safety [5,6,8] which was confirmed by a recent meta-analysis [10]. The only prospective study comparing IA and PE in CIDP [16] reported a good safety profile for both methods and comparable incidences of AEs. One retrospective study reported that both PE and IA were safely applied in 19 patients with GBS [17]. In summary, safety data for PE and IA in neurological diseases largely rely on studies with rather low numbers of subjects, which might explain the large range of reported incidences of AEs as well as the diverging assessments of safety profiles for both methods.

Considering the lack of RCTs regarding the use of PE and IA in neurological diseases, the extensive differences with regard to treatment regimens and peri-procedural measures, and the absence of reliable therapeutic standards for specific disease entities, it is of crucial importance to collect systematic clinical data. In this study, we retrospectively analyzed tolerability and safety data (including adverse events and laboratory abnormalities) in 284 patients (548 treatment cycles, 2470 treatments) with various neurological indications who were treated with either PE (*n* = 65) or IA (*n* = 219) between 2013 and 2020 in our center. We primarily aimed at (1) verifying the advantageous safety and tolerability profile of IA as proposed by previous studies and (2) evaluating our specific PE-regimen which features a comparatively low PV treated per session (0.7-fold) compared to previous publications, allowing daily treatments. 

## 2. Methods

### 2.1. Patients

All patients who were treated with either PE or IA between 2013 and 2020 in the Department of Neurology, University of Ulm, were analysed. All clinical information including medical history, neurological status, adverse events, laboratory data, and clinical scales were collected by reviewing the complete medical records of each patient, including discharge letters, diagnostic findings, and monitoring documents. We included patients with all neurological diagnoses who received at least one treatment of PE or IA. Overall, 284 patients (65 PE, 219 IA) were identified. Because some patients received more than one cycle, 548 cycles (113 PE, 435 IA) were performed and analysed. Reasons for multiple cycles per patient included chronic diseases like CIDP which necessitate the application of multiple cycles in regular time intervals, or insufficient treatment response. One cycle consisted of 5 treatments, resulting in a total of 2740 treatments (565 PE, 2175 IA) which were separately documented and analysed.

All patients with MS fulfilled the 2017 MacDonald diagnostic criteria for MS [18] or CIS at the time of treatment. Patients with CIDP fulfilled the European Fedaration of Neurological Societies (EFNS) criteria for possible, probable, or definite CIDP. Patients with GBS showed the typical clinical picture including rapidly progressive bilateral limb weakness and sensory deficits, hypo-/areflexia, electrophysiological signs of demyelination, and increased protein levels in cerebrospinal fluid. Patients with other diseases were likewise diagnosed based on the respective internationally accepted guidelines.

### 2.2. Indication for PE/IA

All patients were treated in the Neurological Department of Ulm University, Neurological Center of Apheresis and Therapies (Neurologisches Apherese- und Therapiezentrum, NATZ). The decision to perform PE or IA was based on individual evaluation, taking into account diagnosis, clinical and diagnostic findings, and response to previous treatments. In patients with MS or clinically isolated syndrome (CIS), prerequisite for apheresis was the unsuccessful application of at least one cycle of high-dose intravenous methyl-prednisolone (MP). In cases of incomplete improvement, a second cycle of high-dose intravenous MP was performed in some patients. In CIDP, apheresis was only applied in therapy-refractory cases, i.e., patients who deteriorated despite MP and/or IVIg therapy (usually both). In case of a positive treatment effect, apheresis was applied in regular time intervals, based on the individual course of disease, i.e., PE/IA was performed when symptoms began to worsen again after the initial improvement. In GBS, apheresis was used as a first-line therapy as an alternative to IVIg. In some cases, PE/IA was performed after an initial unsuccessful application of IVIg. In all other indications, apheresis was usually performed as an escalation therapy after unsatisfying response to the first-line/standard therapies. The decision for the specific method (PE or IA) was individually made based on current evidence, personal preference/experience, pathophysiological considerations, and comorbidities/contraindications in a process of shared decision-making after in-depth information of each individual patient about all therapeutic options. In 21 patients (8 MS, 2 CIDP, 3 GBS, and 8 other) PE was switched to IA, and in 22 patients (4 MS, 4 CIDP, 3 GBS, and 11 other) IA was switched to PE after one initial unsuccessful cycle. 

### 2.3. Procedures

PE and IA were both applied on 5 consecutive days. The majority of patients received a Shaldon catheter in the right jugular vein. In patients who received several cycles over a prolonged period of time (mainly CIDP), a cubital arteriovenous shunt was used in a few cases. Heparin and citrate were used as anticoagulants, and no prophylactic antibiotics or post-procedural IVIg were given. Before each treatment, a systemic infection was ruled out by blood and urine analysis, and ACE inhibitors were paused at least 3 days before IA. Patients were extensively informed about risks as well as alternative treatment options and gave their written informed consent. During each treatment, a continuous monitoring was performed including blood pressure, heart rate, and oxygen saturation. Laboratory testing including blood count, CRP, electrolytes, liver, and kidney parameters were routinely done on a daily basis during PE/IA. 

During PE, a fixed PV of 2 L (corresponding to a mean individual 0.7-fold PV) was exchanged until 07/2018; afterwards, we instead exchanged the 0.7-fold individual PV. Since comparative studies regarding different treatment regimens for PE/IA are completely lacking, these parameters are mainly based on local experience and expertise. A COM.TEC cell separator (Fresenius Kabi Deutschland GmbH, Bad Homburg, Germany) was used during PE. 

During IA, the 2.0-fold individual plasma volume was processed on the first day, and the 2.5-fold individual plasma volume was processed on days 2–5. The individual plasma volume was calculated according to the formula published by Sprenger et al. [19]. Three different regenerable double-column adsorbers were used: protein A (Immunosorba, Fresenius Medical Care, Bad Homburg, Germany), Peptid-GAM (Globaffin, Fresenius Medical Care, Bad Homburg, Germany), and recombinant proteins (Miltenyi Biotec, Bergisch Gladbach, Germany). All three adsorbers selectively bind human immunoglobulins while largely sparing other plasma proteins. The choice of adsorber was mainly based on availability. ADAsorb (medicap clinic GmbH, Ulrichstein, Germany) and Life 21 (Miltenyi Biotec, Bergisch Gladbach, Germany) were used as immunoadsorption devices; COM.TEC (Fresenius Kabi Deutschland GmbH, Bad Homburg, Germany) and ART Universal (Fresenius Medical Care, Bad Homburg, Germany) were used for cell separation. 

### 2.4. Outcome Parameters

Adverse events were retrospectively collected by reviewing the medical reports and monitoring curves of each patient and treatment. Laboratory changes were assessed based on daily laboratory reports. Adverse events were classified as Grade 1–5 according to the Common Terminology Criteria for Adverse Events grading scale v5.0.

Efficacy parameters before and after treatment were collected as documented in the medical reports. In patients with MS, these include the Expanded Disability Status Scale (EDSS) and the Multiple Sclerosis Functional Composite (MSFC) as the best validated and frequently used standardized clinical scales. In patients with CIDP, we routinely performed the CIDP score [20], which incorporates the Inflammatory Neuropathy Cause and Treatment (INCAT) score [21], the Oxford muscle strength grading score, and vibration sensitivity testing with a 256-Hz Ryder-Seiffel tuning fork. Since no generally accepted and adequately validated standardized scale exists for GBS, evaluation of efficacy in these patients was based on neurological examination before and after PE/IA and classified as large, partial, equivocal, or no improvement. For other indications, no systematic evaluation of efficacy was done due to low numbers of patients. Efficacy data refer to subgroups of patients with sufficient clinical data and have been published previously [5,20,22].

### 2.5. Statistical Analysis

Adverse events and laboratory changes were evaluated per cycle. Adverse events were additionally analysed on a per-patient basis in order to exclude bias based on the per-cycle approach (i.e., one patient may present one specific AE during multiple cycles, causing an overestimation of this AE). Statistical analysis was based on absolute/relative frequencies (categorial variables) and median/interquartile range (continuous variables). For evaluation of laboratory changes, we calculated the change between baseline and second day of PE/IA (not shown) as well as fifth day of PE/IA (before last treatment); we also recorded the share of cycles/patients with pathological values for each laboratory parameter.

Changes of patient related continuous data were investigated with the Wilcoxon signed rank test. Group comparisons for patient related continuous data were performed using the Mann–Whitney-U-test. Group comparisons for patient related categorical data were carried out with the chi-square test or Fisher’s exact test as appropriate. Group comparisons for cycle related continuous data were investigated using linear mixed effects regression models in order to account for patients receiving multiple cycles. Group comparisons for cycle related binary data were investigated using mixed effects regression models for binary outcomes. 

The level of significance was set as *p* ≤ 0.05 (two-sided). To estimate treatment effects, we calculated median differences including a two-sided 95% confidence interval. Statistical analyses were done using SAS, version 9.4, and GraphPad Prism, version 7.05. Because of the explorative nature of this study, all results from statistical tests have to be interpreted as hypothesis-generating rather than proof of efficacy. No adjustment for multiple testing was done.

## 3. Results

### 3.1. Demographics and Clinical Characteristics

Demographic and clinical characteristics are depicted in Table 1. PE and IA patients were not different with regard to age, sex distribution, and body mass index (BMI). For more detailed clinical information for patients with the most common diagnoses (MS, CIDP, and GBS) see Tables S1–S3. Prognostic factors in patients with MS, CIDP, and GBS were evenly distributed between PE and IA.

### 3.2. Adverse Events

Overall, both methods showed an excellent safety profile; no treatment-associated deaths or life-threatening adverse events were recorded. Severe AEs (corresponding to grade 3 on the Common Terminology Criteria for Adverse Events grading scale) in the IA group included three patients with sepsis, one severe pneumonia, and one pneumothorax, corresponding to 5/435 affected IA cycles (1.1%). In the PE group, no severe AEs were recorded. 

Importantly, all three patients with sepsis were diagnosed with CIDP, two-thirds were older than 80 years and multimorbid. One patient had type 2 diabetes mellitus and recurrent urinary infections in medical history. Primary focus was the Shaldon catheter in all three cases. All patients recovered with antibiotic treatment. One severe pneumonia occurred in a 49 year old female with severe myasthenic crisis who was monitored on intensive care unit and dependent on non-invasive ventilation when IA was initiated; she eventually recovered. The pneumothorax (IA group) was a complication of Shaldon catheter placement and necessitated a Bülau drainage as well as a short stay on intensive care unit. The patient recovered completely and received several IA cycles afterwards without any further complications.

Surprisingly, we found that mild and moderate adverse events per cycle (grade 1 and 2; Table 2) were more frequent in the IA group (67.1%) compared to PE (35.4%; *p* < 0.001). With the exception of fatigue, all adverse events were more frequent in the IA group. Most common were intermittent hypotonia (24.0% of all patients), hematoma caused by Shaldon placement (16.4%), and mild infections (6.9%). All adverse events were uncomplicated and did not necessitate any specific therapy. Thrombotic events included deep venous thromboses, most commonly of the Shaldon-affected jugular vein, which were treated with oral anticoagulants and healed without permanent consequences in all cases. Thrombotic events were most frequently seen in patients with GBS. 

Per-patient analysis yielded similar results as per-cycle analysis (not shown). Albeit CIDP patients were older and had more co-morbidities compared to MS, we did not detect any meaningful disease-specific characteristics with regard to AEs.

### 3.3. Laboratory Changes

Median laboratory changes between the day of last treatment (before last treatment) compared to baseline of each cycle in both groups are displayed in Table 3. While loss of thrombocytes was more pronounced in IA, loss of erythrocytes was more pronounced in PE. IA patients showed larger decreases of potassium and calcium; sodium was rather stable in PE as well as IA. The substitution of proteins masks the assumedly larger loss of plasma proteins in PE compared to IA. Importantly, fibrinogen levels were similar in PE and IA. As described previously, we found that in the IA group IgG was removed more effectively than IgA and IgM; consistently, IgG reduction was more pronounced in IA, while IgA and IgM reduction were more pronounced in PE. Supplementing the absolute changes, Table 4 displays the share of patients above/below the pathological threshold for each laboratory parameter per group which yielded congruent results.

### 3.4. Efficacy

Efficacy data for patients with sufficient standardized data have previously been published. These data refer to subsets of the study population investigated in the current study and were treated with the same IA and PE protocols.

In steroid-refractory MS, we conducted a randomized controlled trial in 61 patients (31 IA vs. 30 PE, 5 treatments on 5 consecutive days as outlined above) [5]. We found a significant improvement of symptoms after four weeks compared to pre-treatment in both groups as measured by MSFC and EDSS. In the PE group, median MSFC improved from 0.22 (–0.27 to 0.55) to 0.57 (0.15 to 0.82; *p* < 0.001), and median EDSS improved from 3.0 (2.0 to 3.5) to 2.0 (1.0 to 3.5; *p* < 0.001). In the IA group, median MSFC improved from 0.09 (–0.19 to 0.39) to 0.63 (0.21 to 0.90; *p* < 0.001), and median EDSS improved from 3.0 (2.0 to 4.0) to 2.0 (1.0 to 3.1; *p* < 0.001). Although improvement started earlier in the PE group, MSFC improvement (0.385 vs. 0.265; *p* = 0.03) and response rates (86.7% vs. 76.7%) after four weeks were larger in the IA group. 

In CIDP, we performed a prospective observational study in 17 patients with therapy-refractory courses (insufficient response to steroids and/or IVIg) who underwent IA [20]. Overall, median CIDP scores improved from 308.0 (266.0 to 374.5) pre-treatment to 330.0 (290.0 to 393.5; *p* = 0.02) after two weeks. Furthermore, we were able to stabilize disease progression in 6/7 patients who received long-term IA treatments in regular intervals. Before IA, these patients lost 6.7 (3.0 to 13.1) points of CIDP score per month, while during IA, they lost 0.1 (0.0 to 0.8) points. Due to the insufficient number of patients treated with PE, we cannot provide any comparative results.

A retrospective analysis of 20 patients with GBS [22] yielded response rates of 61.5% for IA and 71.4% for PE after the last treatment based on the documented neurological examinations.

## 4. Discussion

This study aimed at evaluating safety and tolerability of PE and IA as main options of apheresis in autoimmune-mediated neurological diseases. Pre-existing evidence suggested that IA may be superior to PE in this regard, although comparative studies with high numbers of patients are missing. For this purpose, we analysed data from 284 patients (548 cycles, 2740 treatments) who were treated with either PE or IA between 2013 and 2020 in our center under standardized conditions. Importantly, we used an adjusted protocol for PE, aiming for a comparatively small volume of exchanged plasma volume per day (0.7-fold individual PV per day) compared to other commonly used regimens which imply higher volumes (1.0–1.5-fold individual PV per day). This protocol is based on our own clinical experience, including a randomized controlled trial in MS [5] which suggested an excellent safety and good efficacy profile for this specific regimen. The retrospective nature of this study has to be mentioned as a limitation, since we cannot exclude that AEs occurred or diagnoses changed after discharge. We regard the high number of treatments under standardized, monocentric conditions and the continuous, systematic recording of safety data as strengths of this study.

Overall, we found that both methods were very safe across all neurological indications, since we did not record any life-threatening complications or deaths, and grade 3 AEs were recorded in only 1.1% of IA cycles while in PE, we did not record any grade 3 AEs. Therefore, the incidence of serious AEs was even rarer in PE compared to IA. Surprisingly, we found that the share of mild and moderate AEs, including thrombosis, hypotonia, allergic reactions, nausea, and vegetative symptoms were also lower in PE which contradicts the common conception of IA as a better tolerated method of apheresis. However, a closer look at current literature reveals that this question has not been conclusively answered as highlighted by several studies in MS [6,8] as well as a recent meta-analysis [10] which found similar incidences of adverse events. The generally favourable safety profile of both PE and IA should also be considered when weighing against alternative treatment options, for example whether a second high-dose MP cycle should be performed in steroid-refractory MS relapse before apheresis. As highlighted by a recent publication [23], sparing a second MP cycle and applying apheresis directly may be superior in terms of efficacy and safety. 

Data about complication rates of PE are heterogenous, varying between 4.2% and 25.6% [13,24,25,26], most likely due to heterogenous treatment regimens with regard to PV treated per session, type and dosage of applied anticoagulants, type of venous access (peripheral or central), and type of volume substitution (human albumin or FFP). Basic-Jukic et al. found moderate allergic reactions in 1.6% of 509 patients treated with PE as well as severe anaphylactic reactions in five cases; accordingly, Schneider-Gold et al. found a higher incidence of allergic reactions in patients with myasthenia gravis treated with PE compared to IA [11]. However, the incidence of allergic reactions in PE may presumably be lowered by using human albumin solution instead of FFP, since allergic reactions are commonly associated with FFP [27], but very rarely with human albumin solution [28]. Accordingly, the incidence of allergic reactions was extremely low in our PE study population (0.8%), and lower compared to IA (4.6%). Since no volume substitution is needed in IA, the higher incidence of allergic reactions in IA may be associated with the higher amounts of anticoagulants needed for IA, especially heparin. Furthermore, it cannot be ruled out completely that adsorber substances may be reinfused.

Importantly, in order to utilize the advantageous safety profile of human albumin solution compared to FFP in PE, it is essential to limit fibrinogen loss by either reducing the frequency of treatments (i.e., performing treatments on a two-day instead of a daily basis) or reducing the PV processed during each treatment. Based on our data, it was possible to perform daily PE treatments with 0.7-fold PV without any treatment interruptions while maintaining acceptable fibrinogen plasma levels, i.e., fibrinogen levels remained above 0.8 g/L before each treatment. Applying this scheme, we found comparable fibrinogen levels in PE and IA, suggesting that (1) fibrinogen loss in PE can be sufficiently controlled by attenuating PV per treatment, and (2) significant loss of fibrinogen is also present in IA. In line with our finding of similar and acceptable fibrinogen levels in both PE and IA, we did not record any bleeding complications with both measures, while the incidence of thrombotic events was similar (2.7% in PE, 3.4% in IA). Therefore, we can conclude that maintaining higher fibrinogen levels during PE may contribute to improve safety, since bleeding complications have previously been described to occur more frequently in PE (3.1% of treatments) compared to IA (1.3%) [15].

In addition to allergic reactions and bleeding complications, infections induced by the immune-modulating effects of PE/IA are a major concern. Based on our data, both measures were very safe in this regard. We found severe infections with consecutive sepsis in three patients who were all diagnosed with CIDP; two of them were >80 years old. Therefore, we conclude that these clinical characteristics constitute risk factors which have to be considered. Mild infections occurred in only 5.3% (PE) and 7.4% (IA), respectively. These data corroborate the conception that peri-procedural prophylactic application of antibiotics or immunoglobulins is not needed. We could not confirm the finding of Schneider-Gold et al. who found a higher frequency of respiratory infections in PE compared to IA [11]. Again, different treatment regimens (0.7-fold vs. up to 1.5-fold PV treated in PE patients) do most likely account for this discrepancy, since treating lower PVs per session implies a higher preservation of antibodies and other anti-infective plasma proteins. Accordingly, we found that overall reduction rates of immunoglobulins were about similar in PE and IA; regarding subclasses, we found that reduction rates of IgA and IgM were higher in PE, while reduction rate of IgG was higher in IA. This was expected since the applied IA adsorbers feature a higher IgG affinity [29].

Regarding mild adverse events such as hypotonia, nausea, and palpitations, we generally found higher incidences in the IA group. This finding is not necessarily caused by technical differences of PE and IA, but may simply be explained by the significantly prolonged treatment times in IA. Applying the treatment regimens outlined above and dependent on the patient’s individual PV, one IA treatment requires about the double amount of time compared to PE due to the excessive amount of blood treated during each session. Apart from the higher incidence of adverse events which are associated with longer treatment times, this also implies a larger burden for the patient.

Subclinical laboratory changes were unproblematic in all cases and did not necessitate specific treatment, with the exception of potassium and protein substitution which are routinely done in PE as well as IA. Interestingly, we found a higher incidence of anemia in PE, but a higher incidence of thrombopenia in IA, confirming previous findings [5]. The latter may possibly be explained by the higher demand for heparin during IA, since the external blood circuit has to be maintained for a longer timeframe and heparin may cause heparin-induced thrombopenia (HIT). While cell count abnormalities can be contributed to the procedures themselves, changes of plasma constituents like liver transaminases or urea do not necessarily imply organic disturbances, but rather signify that these substances are removed by the procedures. Regarding electrolytes, hypokalemia and hypocalcemia (due to citrate binding) are both known phenomena in PE and IA. In our study electrolyte disturbances were more frequent in the IA group. 

Despite the lower incidence of AEs and laboratory abnormalities found for the low-PV PE treatment regimen compared to IA, this finding has to be interpreted carefully because of the following limitations of this study: First, the safety profile was compared with IA, which is quite a novel therapeutic approach itself, especially with regard to neurological diseases. A superiority of our PE regimen compared to other commonly applied regimens can however only be proven by conducting a direct comparative prospective study. Secondly, lowering the PV treated each day may compromise the efficacy of the procedure, which cannot be adequately analysed by means of a retrospective study design. We investigated the efficacy of this approach in a randomized controlled study in patients with steroid-refractory MS relapse versus IA [5]. Indeed, we found that after four weeks, IA patients showed a significantly larger improvement of the MSFC; however, the difference between PE and IA was rather small.

In summary, we conclude that:

1. PE and IA constitute safe and generally well-tolerated therapeutic options in autoimmune-mediated neurological diseases.
2. Contrary to previous publications, we found a lower incidence of adverse events in the PE group—possibly due to the low-volume per treatment regimen (0.7-fold PV per day), which allows to use human albumin solution while maintaining sufficient fibrinogen levels.
3. Safety and efficacy of this specific PE treatment regimen have to be further evaluated by means of a directly comparative, prospective study.
4. This study highlights the importance to consider specific treatment regimens with regard to safety and efficacy in general when assessing apheresis studies.

## Figures and Tables

**Table 1:** Baseline Characteristics.

	IA	PE	Total	*p*
Patients (cycles)	219 (435)	65 (113)	284 (548)	
Treatments per cycle	5	5		
Processed PV per treatment	2.0–2.5-fold	0.7-fold		
Age (years)	51.0 (36.0 to 62.0)	45.5 (34.5 to 63.0)	50.0 (36.0 to 62.0)	0.68
Sex				0.89
male	94 (42.9%)	27 (41.5%)	121 (42.6%)	
female	125 (57.1%)	38 (58.5%)	163 (57.4%)	
BMI (kg/m²)	24.3 (21.8 to 27.8)	25.2 (21.6 to 27.5)	24.6 (21.8 to 27.8)	0.67
Diagnosis				
MS	72 (32.9%)	21 (32.3%)	93 (32.7%)	
CIS	28 (12.8%)	10 (15.4%)	38 (13.4%)	
NMOSD	3 (1.4%)	2 (3.1%)	5 (1.8%)	
AE	15 (6.8%)	2 (3.1%)	17 (6.0%)	
CIDP	30 (13.7%)	4 (6.2%)	34 (12.0%)	
GBS	17 (7.8%)	10 (15.4%)	27 (9.5%)	
MG	4 (1.8%)	0	4 (1.4%)	
SPS	3 (1.4%)	1 (1.5%)	4 (1.4%)	
SLE	1 (0.5%)	2 (3.1%)	3 (1.1%)	
Other	46 (21.0%)	12 (18.4%)	58 (20.4%)	

IA—immunoadsorption; PE—plasma exchange; PV—individual plasma volume; BMI—body mass index; MS—multiple sclerosis; CIS—clinically isolated syndrome; NMOSD—neuromyelitis optica spectrum disorder; AE—autoimmune encephalitis; CIDP—chronic inflammatory demyelinating polyneuropathy; GBS—Guillain–Barré syndrome; MG—myasthenia gravis; SPS—stiff person syndrome; SLE—systemic lupus erythematodes.

**Table 2:** Adverse Events per Cycle.

	MS		CIDP		GBS		Overall		
Adverse Event	PE (*n* = 27)	IA (*n* = 100)	PE (*n* = 18)	IA (*n* = 80)	PE (*n* = 15)	IA (*n* = 22)	PE (*n* = 113)	IA (*n* = 435)	Total (*n* = 550)
Hypotonia	7.4	23.0	5.6	16.3	0.0	31.8	8.8	28.0	24.0
Hematoma (Shaldon)	3.7	18.0	16.7	6.3	13.3	9.1	11.5	17.7	16.4
Mild Infections	0.0	4.0	5.6	7.5	20.0	13.6	5.3	7.4	6.9
Technical Complications	0.0	6.0	0.0	7.5	0.0	13.6	0.8	5.7	4.7
Nausea	3.7	3.0	0.0	1.3	0.0	9.1	3.5	4.8	4.5
Tachycardia	0.0	3.0	0.0	7.5	13.3	4.5	1.8	4.6	4.0
Edema	0.0	3.0	0.0	3.8	0.0	4.5	0.0	4.8	3.8
Allergic Skin Reaction	0.0	10.0	5.6	2.5	0.0	4.5	0.8	4.6	3.8
Thrombosis	3.7	0.0	0.0	3.8	6.7	13.6	2.7	3.4	3.3
Thoracic Pain	0.0	3.0	0.0	1.3	0.0	13.6	0.8	3.9	3.3
Fatigue	0.0	0.0	5.6	1.3	6.7	0.0	3.5	1.1	1.6
Thrombosis	3.7	0.0	0.0	3.8	6.7	13.6	2.7	3.4	3.3

Data are %. Table presents all adverse events that occurred in >3% of patients in at least one of the treatment groups. IA—immunoadsorption; PE—plasma exchange; MS—Multiple Sclerosis; CIDP—Chronic Inflammatory Demyelinating Polyneuropathy; GBS—Guillain–Barré syndrome.

**Table 3:** Laboratory Changes.

Parameter	PE (*n* = 113)		IA (*n* = 435)		*P*
Leukocytes (G/L)	–0.3	(–1.6 to 0.9)	0.1	(–1.4 to 1.3)	0.98
Erythrocytes (T/L)	–0.55	(–0.72 to –0.21)	–0.35	(–0.59 to –0.14)	**<0.001**
Hemoglobin (g/L)	–15	(–23 to –8)	–10	(–17 to –4)	**<0.001**
Hematocrit (%)	–4	(–2 to –6)	–3	(–1 to –5)	**<0.001**
Thrombocytes (G/L)	–53	(–90 to –26)	–91	(–128 to –55)	**<0.001**
MPV(fL)	0.1	(–0.3 to 0.4)	0.4	(0.1 to 0.8)	**<0.001**
Quick (%)	–24	(–37 to –12)	–10	(–39 to 2)	0.18
INR	0.13	(0.07 to 0.44)	0.06	(–0.01 to 0.37)	0.66
pTT (s)	11.6	(4.6 to 32.3)	6.5	(3.1 to 26.0)	0.52
Fibrinogen (g/L)	–1.7	(–1.9 to –0.1)	–1.6	(–3.4 to –0.8)	0.96
Sodium (mmol/L)	1	(–1 to 3)	2	(1 to 4)	**0.003**
Potassium (mmol/L)	–0.23	(–0.60 to 0.08)	–0.47	(–0.77 to –0.17)	**<0.001**
Calcium (mmol/L)	0.02	(–0.08 to 0.10)	–0.17	(–0.25 to –0.06)	**<0.001**
Urea (mmol/L)	–0.12	(–0.98 to 0.76)	–0.94	(–2.14 to 0.05)	**0.019**
Creatinine (µmol/L)	–1.5	(–8.5 to 1.8)	2	(–5.5 to 8)	**0.002**
GFR (ml/min)	2.0	(–3.5 to 10.5)	–2.5	(–13 to 7.3)	**0.002**
AST (U/L)	9.0	(–6.25 to 13.8)	1.0	(–4.0 to 10.0)	0.98
ALT (U/L)	–6.0	(–13.0 to 7.0)	–3.5	(–9.3 to 9.0)	0.85
GGT (U/L)	–17.0	(–27.5 to –12.0)	–9.0	(–22.0 to –4.0)	0.92
AP (U/L)	–39.0	(–43.0 to –26.0)	–19.0	(–30.3 to –12.0)	**0.03**
Bilirubin (µmol/L)	3.5	(0.5 to 5.3)	0.1	(–2.4 to 11.4)	0.09
Protein (g/L)	–5.9 *	(–9.4 to –1.8)	–19.9	(–23.9 to –15.8)	**<0.001**
CRP (mg/L)	0.08	(–1.02 to 1.40)	0.81	(–0.28 to 3.81)	0.23
IgA (mg/L)^#^	–866	(–572 to –1100)	–1362	(–1197 to –1536)	**<0.001**
IgG (mg/L)^#^	–6639	(–5428 to –7367)	–5770	(–5562 to –6582)	**<0.001**
IgM (mg/L)^#^	–711	(–316 to –924)	–713	(–324 to –1066)	**0.008**

Median laboratory changes (IQR) between the day of last treatment compared to baseline of each cycle in both groups. IA—immunoadsorption; PE—plasma exchange; MPV—mean platelet volume; INR—international normalized ratio; pTT—partial thromboplastin time; GFR—glomerular filtration rate; AST—aspartate aminotransferase; ALT—alanine aminotransferase; GGT—gamma glutamyl transferase; AP—alkaline phosphatase; CRP—C-reactive protein; * after substitution with human albumin solution; ^#^ Immunoglobulins A, G, and M were measured in a subset of 61 MS patients who participated in a randomized controlled study [5]. Bold *p*-values mark significant values.

**Table 4:** Pathological Laboratory Parameters at Last Day of Apheresis (Before Last Treatment).

Parameter	PE (*n* = 113)	IA (*n* = 435)	*p*
Leukocytes (G/L)	18.3%	19.0%	0.64
Erythrocytes (T/L)	53.9%	38.5%	**0.05**
Hemoglobin (g/L)	58.7%	46.0%	0.08
Hematocrit (%)	54.8%	45.8%	0.09
Thrombocytes (G/L)	12.6%	41.5%	**<0.001**
MPV(fL)	4.9%	6.1%	0.46
Quick (%)	44.8%	37.5%	0.15
INR	90.0%	91.0%	0.82
pTT (s)	78.6%	79.9%	0.85
Fibrinogen (g/L)	80.0%	66.7%	0.82
Sodium (mmol/L)	5.8%	0.3%	0.13
Potassium (mmol/L)	9.7%	27.0%	**0.001**
Calcium (mmol/L)	5.2%	17.8%	0.11
Urea (mmol/L)	8.3%	7.1%	0.90
Creatinine (µmol/L)	23.4%	28.1%	0.80
GFR (ml/min)	85.3%	82.6%	0.42
AST (U/L)	33.3%	23.8%	0.07
ALT (U/L)	18.2%	20.6%	0.49
GGT (U/L)	0.0%	15.7%	0.15
AP (U/L)	100.0%	88.5%	0.64
Bilirubin (µmol/L)	16.7%	11.4%	0.52
Protein (g/L)	92.0%*	99.1%	**0.004**
CRP (mg/L)	24.5%	40.3%	0.23

Share of patients with values below or above the pathological threshold for each parameter at last day of apheresis in each group. IA—immunoadsorption; PE—plasma exchange; MPV—mean platelet volume; INR—international normalized ratio; pTT—partial thromboplastin time; GFR—glomerular filtration rate; AST—aspartate aminotransferase; ALT—alanine aminotransferase; GGT—gamma glutamyl transferase; AP—alkaline phosphatase; CRP—C-reactive protein; * after substitution with human albumin solution. Bold *p*-values mark significant values.

## Supplementary Materials

The following are available online at https://www.mdpi.com/2077-0383/9/9/2874/s1, Supplementary Table S1: Baseline Characteristics of patients with MS/CIS; Supplementary Table S2: Baseline Characteristics of patients with CIDP; Supplementary Table S3: Baseline Characteristics of patients with GBS.
