# Supplementary material for: Safety and Tolerability of Plasma Exchange and Immunoadsorption in Neuroinflammatory Diseases
Source: J Clin Med. 2020 Sep 5;9(9):2874. doi: 10.3390/jcm9092874 (PMC7565027; doi:10.3390/jcm9092874)
Supplement: Supplementary file 1 [file jcm-09-02874-s001.pdf]

Supplementary Table 1. Baseline Characteristics of patients with MS/CIS

	IA	PE	Total	p
**Patients (cycles)**	72 (100)	21 (27)	93 (127)	
**Age (years)**	41.5 (29.0–49.0)	38.0 (29.0–40.5)	40.0 (29.0–49.0)	0.23
**Sex**<br>male<br>female	 19 (26.4 %)<br>53 (73.6 %)	 5 (23.8 %)<br>16 (76.2 %)	 24 (25.8 %)<br>69 (74.2 %)	1.00
**BMI (kg/m²)**	24.0 (21.2–27.9)	23.9 (21.0–26.4)	23.9 (21.3–27.6)	0.74
**Age at Onset (years)**	28.5 (23.8–39.0)	29.0 (17.0–37.0)	29.0 (22.5–38.0)	0.71
**Age at Diagnosis (years)**	30.5 (25.0–39.3)	29.5 (22.0–39.0)	30.0 (25.0–39.0)	0.70
**Time between Relapse and IA/PE (days)**	70 (36–136)	46 (11–375)		0.88
**Disease-modifying Therapies**				
Interferon	16 (22.2%)	2 (9.5%)	18 (19.4%)	
Glatiramer acetate	12 (16.7%)	2 (9.5%)	14 (15.1%)	
Fingolimod	16 (22.2%)	2 (9.5%)	18 (19.4%)	
Natalizumab	8 (11.1%)	2 (9.5%)	10 (10.8%)	
Teriflunomid	5 (6.9%)	1 (4.8%)	6 (6.5%)	
Dimethyl fumarate	5 (6.9%)	3 (14.3%)	8 (8.6%)	

IA – immunoadsorption; PE – plasma exchange; BMI – body mass index

Supplementary Table 2. Baseline Characteristics of patients with CIDP

	IA	PE	Total	p
**Patients (cycles)**	30 (80)	4 (18)	34	
**Age (years)**	61.5 (54.8–71.5)	78.0 (61.0–79.0)	62.0 (55.0–74.0)	0.15
**Sex**<br>male<br>female	 21 (70.0%)<br>9 (30.0%)	 4 (100.0%)<br>0 (0.0%)	 25 (73.5 %)<br>9 (26.5 %)	0.55
**BMI (kg/m²)**	26.2 (24.1–30.9)	24.8(22.6–43.1)	26.1 (23.8–30.9)	0.69
**Age at Onset (years)**	53.0 (46.3–64.8)	61.5 (60.0–63.0)	54.5 (48.8–64.0)	0.33
**Age at Diagnosis (years)**	54.0 (48.0–67.0)	70.0 (70.0–79.0)	60.0 (50.8–70.8)	0.08
**Previous Therapies**<br>Prednisolone<br>IVIg<br>Azathioprine<br>Methotrexate	 20 (66.7%)<br>24 (80.0%)<br>3 (10.0%)<br>1 (3.3%)	 3 (75.0%)<br>3 (75.0%)<br>0 (0.0%)<br>0 (0.0%)	 23 (67.6%)<br>27 (90.0%)<br>3 (8.8%)<br>1 (2.9%)	

IA – immunoadsorption; PE – plasma exchange; BMI – body mass index; IVIg – intravenous immunoglobulines

Supplementary Table 3. Baseline Characteristics of patients with GBS

	IA	PE	Total	p
**Patients (cycles)**	17 (22)	10 (15)	27	
**Age (years)**	52.0 (36.0–63.0)	66.0 (60.5-73.0)	61.0 (39.0–70.5)	0.07
**Sex**<br>male<br>female	 7 (41.2%)<br>10 (58.8%)	 4 (40.0%)<br>6 (60.0%)	 11 (40.7 %)<br>16 (59.3 %)	1.00
**BMI (kg/m²)**	22.3 (21.3–25.2)	27.0 (19.5–29.2)	23.5 (21.2–26.6)	0.12

IA – immunoadsorption; PE – plasma exchange; BMI – body mass index; IVIg – intravenous immunoglobulines
